# Pneumoperitoneum Due to Sexual Intercourse Status Post-Salpingo-Oophorectomy Secondary to Sex, Cunnilingus, and Air-Insufflating Vibrator

**DOI:** 10.7759/cureus.79584

**Published:** 2025-02-24

**Authors:** Anupam K Gupta

**Affiliations:** 1 General Surgery, SSM (Sisters of St. Mary) Health Good Samaritan Hospital, Mt Vernon, USA

**Keywords:** air insufflating vibrator, cunnilingus, intercourse, pneumoperitoneum, sex

## Abstract

Pneumoperitoneum and its related abdominal pain are usually a surgical emergency that dictates ruling out a perforated viscus. There are multiple causes of pneumoperitoneum, which are nonsurgical; we report a case of a patient after salpingo-oophorectomy for ectopic pregnancy presenting in the postoperative period after a week with excess pneumoperitoneum due to sexual intercourse, cunnilingus, and use of air insufflating vibrator.

## Introduction

Pneumoperitoneum with abdominal pain is a concerning combination needing surgery in many situations. There are multiple situations where a pneumoperitoneum is present for nonsurgical reasons [1-3]. It is essential to note that air in the peritoneum can cause pain, mimicking peritonitis [4,5].

## Case presentation

**Figure 1:**
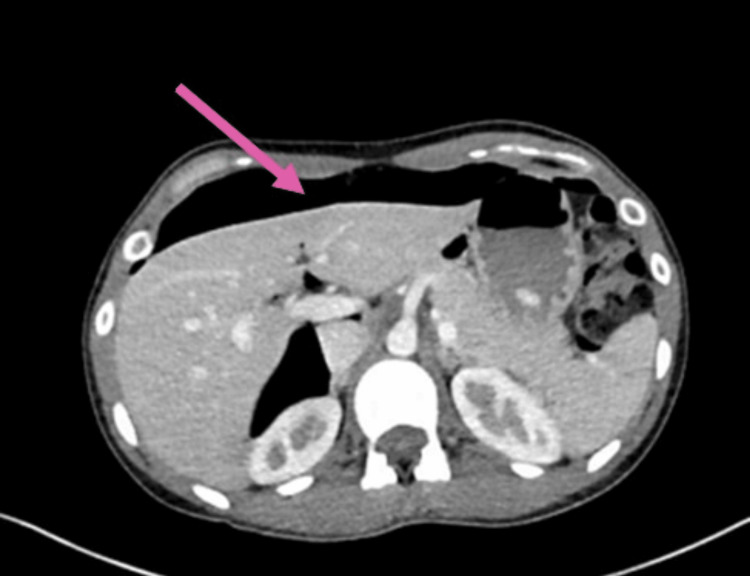
Computed tomography showing pneumoperitoneum, air marked with a pink arrow.

This report describes the case of a 29-year-old lady with a body mass index of 18.25 kg/m^2^. She had a history of drug abuse with methamphetamines, cocaine, and cannabis and a past medical history significant for anxiety. She underwent an emergency laparoscopic right-side salpingo-oophorectomy for ectopic pregnancy. After this surgery, her postoperative course was uneventful, and the patient went home on postoperative day 2. She presented to the emergency room on postoperative day 9 after her right salpingo-oophorectomy with severe abdominal pain and distention. At the time of her arrival, she had diffuse abdominal pain with no specific aggravating or relieving factors. Clinical examination revealed tenderness on all quadrants on deep palpation. Her vital signs, heart rate, blood pressure, and respiratory rate were largely within normal range with a baseline heart rate of about 90 beats per minute, and her blood work, which involved a complete blood profile, was within normal limits with a white blood cell count of 8400 cells/microliter. Computed tomography using intravenous contrast and no oral contrast revealed extensive pneumoperitoneum with no identifiable etiology (Figure 1).

Given the significant size of the pneumoperitoneum and abdominal pain, there was a concern about a viscus perforation or a missed iatrogenic perforation. The patient was taken emergently to the operating room, and a thorough evaluation on diagnostic laparoscopy, subsequently converted to open exploration of the gastrointestinal tract, did not reveal any obvious pathology. The prior surgical site of the right salpingo-oophorectomy has expected post-op hematoma and adhesive appearance with a small amount of hematoma in the vicinity. An intraoperative esophagogastroduodenoscopy and a postoperative gastrografin small bowel follow-through study showed no leak. A discussion with the patient and her partner later revealed she was involved in sexual activity, which involved cunnilingus and the use of a vibrator that used pulse air to provide sexual stimulation before the onset of her symptoms on the day they came to the emergency room. Her postoperative course was uneventful, and she was sent home on postoperative day 4 from her second surgery.

## Discussion

Acute abdominal pain with evidence of free air is usually considered a surgical emergency of gastrointestinal origin. The predominant cause of pain is irritation of the peritoneum with bowel contents or inflammatory response [1-3].

Air irritates the pneumoperitoneum and elicits pain [4]. Multiple cases of peritoneal irritation with air exist, leading to negative surgical intervention [5,6]. The literature suggests that exposure to air in the peritoneum causes systemic and local inflammatory responses [4,5]. Air is also more inflammatory to the peritoneum than carbon dioxide [6,7]. 

Carbon dioxide used during laparoscopic surgery is eliminated by exhalation much faster, usually within 3-5 days, than room air, which may persist longer [5-7]. The presence of a significant amount of air after a week in our patient was concerning a bowel injury. A computed tomography scan is sensitive to detect pneumoperitoneum [5,6]. 

There is an open communication between the vagina and the abdominal cavity via the fallopian tubes [6-8]. This communication was used to perform a diagnostic test in the past wherein carbon dioxide would be insufflated through the vagina to assess for the patency of fallopian tubes, as a workup of infertility would often lead to pain [6-8]. Cunnilingus, with the use of an air-insufflating vibrator during sexual intercourse, can unknowingly lead to the insufflation of a significant amount of air [6-8]. 

There have been multiple reported cases of pneumoperitoneum via the genital tract, like post-knee chest exercise, vaginal douching, and sexual activity [5-9]. In our patient, a salpingo-oophorectomy likely provided better access to the vagina to the abdominal cavity, given a shorter fallopian tube on one side [9]. Cunnilingus, with air insufflation, can cause fatal air embolism in pregnant ladies in specific reports [10]. In our patient, a combination of sexual intercourse, cunnilingus, and the use of an insufflating vibrator led to significant pneumoperitoneum and symptoms. Her postoperative course was uneventful, and she was sent home on postoperative day 4 from her second surgery. 

## Conclusions

It is essential to differentiate operative and nonoperative courses of pneumoperitoneum. Sexual activity, cunnilingus, and the use of air insufflation vibrators can be a cause of pneumoperitoneum and peritoneal symptoms.
